# Expression and distribution of the transient receptor potential cationic channel ankyrin 1 (TRPA1) in the human seminal vesicles

**DOI:** 10.1002/hsr2.987

**Published:** 2022-12-11

**Authors:** Harrina E. Rahardjo, Stefan Ückert, Markus A. Kuczyk, Petter Hedlund

**Affiliations:** ^1^ Department of Urology, Faculty of Medicine, Cipto Mangunkusumo Hospital University of Indonesia Jakarta Indonesia; ^2^ Department of Urology & Urological Oncology, Hannover Medical School Division of Surgery Hannover Germany; ^3^ Department of Clinical Pharmacology, Faculty of Medicine Linköping University Linköping Sweden

**Keywords:** human seminal vesicles, immunohistochemistry, molecular biology, transient receptor potential cation channel ankyrin 1

## Abstract

**Background and Aims:**

The transient receptor potential cationic channel ankyrin 1 (TRPA1), a channel protein permeable to most divalent cations, has been suggested to play a role in mechano‐afferent/efferent signaling (including the release of neurotransmitters) in the human urinary tract (bladder, prostate, and urethra). To date, only a few studies have addressed the expression of this receptor in male and female reproductive tissues. The present study aimed to evaluate human seminal vesicles (SVs)  for the expression and localization of TRPA1.

**Methods:**

SV tissue was obtained from 5 males who had undergone pelvic surgery due to malignancies of the prostate or urinary bladder. The expression of messenger ribonucleic acid (mRNA) specifically encoding for the TRPA1 protein was elucidated by means of reverse transcriptase polymerase chain reaction (RT‐PCR). Using immunohistochemical methods, the distribution of TRPA1 was examined in relation to the endothelial and neuronal nitric oxide synthases (eNOS, nNOS) and the neuropeptides calcitonin gene‐related peptide (CGRP) and vasoactive intestinal polypeptide (VIP).

**Results:**

RT‐PCR revealed signals related to the expected molecular size of 656 bp. Immunohistochemistry demonstrated that TRPA1 is located in nerves running through the smooth muscle portion of the SV. Here, the protein is in part co‐localized with nNOS and CGRP, whereas no co‐localization with VIP was registered. Dot‐like signals specific for TRPA1 were observed in the cytoplasm of epithelial cells lining the lumen of glandular spaces. The epithelial layer also presented staining for eNOS. The smooth musculature appeared free of immunosignals for TRPA1.

**Conclusion:**

The results convincingly show the expression of TRPA1 in nerve endings as well as in epithelial cells of the SV. Based on its location in epithelial cells, TRPA1 might be involved in the mechanism of the NO/cyclic guanosine monophosphate (GMP)‐mediated signaling and also the control of secretory function (mediated by cyclic GMP) in the human SV.

## 1 INTRODUCTION

The seminal vesicles (SVs) are important organs involved in the process leading up to seminal emission and ejaculation in males. There is extensive evidence that the normal function of SV smooth muscle contributes to the facilitation of seminal emission.^1^ Hence, ejaculatory dysfunction, including premature ejaculation and anejaculation, has been suggested as the possible result of disturbances on the level of neuromuscular control.^2^, ^3^ Nevertheless, in contrast to the knowledge of the pharmacology of the penile erectile tissue, the multiple biochemical signals and interactions mediating the control of the human SV are still poorly understood. To date, several studies have investigated the potential involvement of pathways mediated by alpha‐adrenoceptors, the serotoninergic system, the cyclic AMP and nitric oxide (NO)/cyclic guanosine monophosphate (GMP) pathways, potassium channels, and Rho‐kinase (ROK).^4^, ^5^, ^6^, ^7^, ^8^ The transient receptor potential cationic channel ankyrin 1 (TRPA1) belongs to the superfamily of polymodally gated transient receptor potential (TRP) channels (TRPA, TRPC, TRPM, TRPP, TRPML, TRPV) and has been shown in afferent nerve fibers, capsaicin‐sensitive primary sensory neurons and (uro)epithelial cells in various organs of the urogenital system. It is assumed to be involved in nociception, mechano‐sensory transduction (smooth muscle contraction), and neuronal control including the release of neurotransmitters. The channel protein can be activated by physical stimuli, such as noxious cold, or chemical activators, including arachidonic acid and isothiocyanates, and also some natural stinging compounds from plants (cinnamonaldehyde, allicin, diallyl disulfide).^9^ TRPA1 can act as a molecular sensor of mechanical stimuli (such as stretch, leading to the activation of Ca^2+^ or release of ATP), noxious chemical irritation, and inflammatory processes in the lower urinary tract (urinary bladder, prostate) as well as in male and female genital tissues (corpus cavernosum penis, clitoris, labia minora, vagina).^10^, ^11^, ^12^, ^13^ Since disruptions to sensory pathways that underlie the neuronal control may play a crucial role in the pathophysiology of dysfunctions of lower urinary tract organs, the channel has been proposed as a potential drug target for the symptomatic and/or curative treatment of urinary symptoms (e.g., lower urinary tract symptoms [LUTS] secondary to benign prostatic hyperplasia, overactive bladder [OAB]/detrusor overactivity) and sexual dysfunctions in both sexes. With regard to the ejaculatory response, an association has been postulated between the activity of TRP channels located in the epithelial layer of the glans penis, acting as physiological receptors of physical sensations such as movement of the penis and vaginal humidity, with the condition of lifelong premature ejaculation.^14^ Since, to the best of our knowledge, no study has yet investigated TRPA1 in SVs, our study aimed to determine by means of molecular biology (reverse transcriptase polymerase chain reaction [RT‐PCR]) and conventional immunohistochemistry the expression and distribution of TRPA1.

## 2 MATERIAL AND METHODS

### 2.1 Tissue source and handling

In accordance with the Declaration of Helsinki of the World Medical Association, the guidelines of the local Ethics Committee, and in consent with the regulations of the Division of Surgery of the Hannover Medical School, (non‐tumorous) SV tissue was obtained from five male subjects who had undergone pelvic surgery due to localized malignancies of the prostate or urinary bladder. Histological examination by an expert pathologist of tissue specimens did not show any malignant transformations, such as intraepithelial neoplasia or carcinoma. The tissue was immediately placed in an ice‐cold (+4°C) solution of CUSTODIOL (Dr. Franz Köhler Chemie GmbH) until further handling in the experiments.

### 2.2 RT‐PCR analysis

To investigate by means of RT‐PCR the expression of messenger ribonucleic acid (mRNA) specifically encoding for TRPA1 (hTRPA01FWD 985–1003, hTRPA02REV 1641–1623), tissue portions of the SV were frozen in liquid nitrogen. Reaction mixtures were prepared using up to 5 mg RNA extracted from the specimens, 1 ml desoxy ribonucleoside triphosphate (10 mM), 1 ml oligo (dT) 12–18 (0.5 mg/ml) and made up to 10 ml with sterile water. 0.2 ml were transferred into test tubes and incubated for 5 min at 65°C, followed by placement on ice (for 1 min). A mixture of 2 ml buffer, 4 ml 25 mM MgCl_2_, 2 ml 0.1 M dithiothreitol, and 1 ml recombinant ribonuclease inhibitor (RaceOUT, 40 units/ml) was added followed by incubation for 2 min at 42°C. Fifty units of SuperScript II reverse transcriptase (Invitrogen GmbH) were added to each tube (except for the negative control) followed by incubation for 50 min at 42°C. The reaction was terminated by heating for 15 min to 70°C followed by cooling to 0°C. Two units of RNase H were added to each tube followed by incubation for 20 min at 37°C. PCR amplification was performed using a 1–2 µl complementary DNA (cDNA) template (200 ng/ml), 1–2 µl primers (10 pmol/ml), and 25 µl Taq PCR Master Mix (Qiagen AG). A negative control (without template cDNA) was included in each experiment. PCR analysis was performed in a thermocycler (Model 9600; PE Biosystems GmbH). Cycling conditions were set as follows: denaturation for 3 min at 94°C, 35 cycles of denaturation (30 s at 94°C), annealing phase (30 s at 52–60°C), extension (1 min at 72°C), final extension (10 min at 72°C).^13^, ^14^ Densitometric analysis of the bands was performed using Image J (National Institutes of Health). In the experiments, total mRNA isolated from human prostate tissue served as a reference for the expression of sequences encoding for TRPA1. Messenger RNA for smooth muscle alpha‐actin was used as an internal control for each tissue.

### 2.3 Immunohistochemistry

Following immersion‐fixation for 4 h in 4% formaldehyde in phosphate‐buffered saline (pH 7.4), tissues were rinsed several times with phosphate‐buffered saline containing 15% (w/w) sucrose and then embedded in Tissue‐Tec (Miles Laboratories). Using a cryostat, tissue specimens were sliced to 8–10 µm thickness and thaw‐mounted onto glass slides. Preincubation of sections was done for 2 h in phosphate‐buffered saline with 0.2% Triton X‐100 and 0.1% bovine serum albumin. The sections were then exposed to the primary antibodies (ABs) directed against TRPA1 (working dilution 1:500), calcitonin gene‐related peptide (CGRP, 1:500), neuronal nitric oxide synthase (nNOS, 1:1.000), endothelial NOS (eNOS) and vasoactive intestinal polypeptide (VIP) for 24 h. The sections were rinsed and incubated for 90 min with Alexa Fluor‐conjugated secondary ABs (1:800). Thereafter, sections were mounted in phenylendiamine, and visualization was done using a laser fluorescence microscope (Olympus Corp.).^12^ Images were created using the ViewFinder program (Pixera). Negative controls without the primary ABs were also performed.

### 2.4 AB source

ABs directed against TRPA1 (raised in rabbits) were purchased from Alamone Labs, the mouse anti‐eNOS ABs were from Sigma Chemical, anti‐CGRP and ‐VIP ABs (raised in guinea‐pigs) from EuroDiagnostica, the anti‐nNOS ABs (raised in sheep) were generously provided by Dr. Piers Emson (The Babraham Institute of the Medical Research Council, Department of Neurobiology, Molecular Neuroscience Group). Alexa Fluor ABs were obtained from Molecular Probes Europe BV.

## 3 RESULTS

### 3.1 RT‐PCR analysis

**Figure 1:**
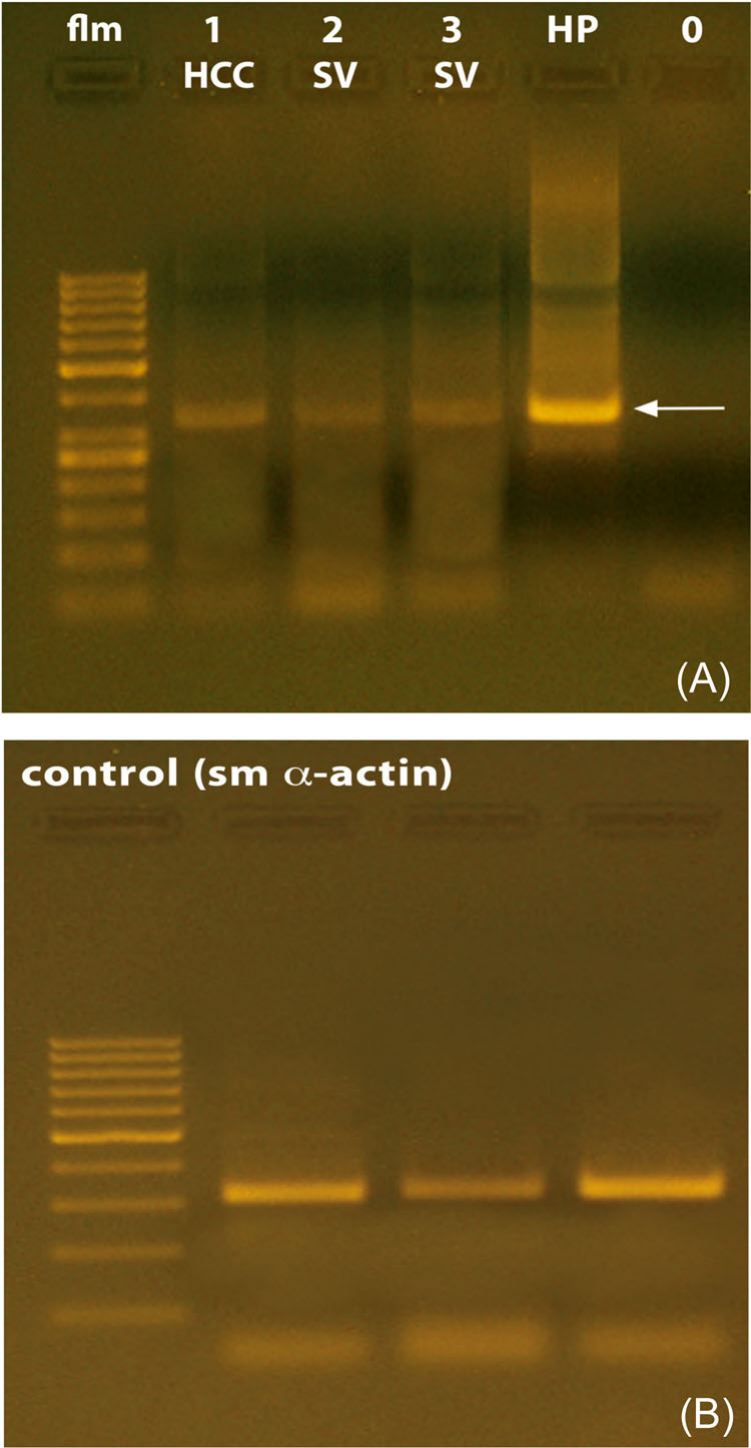
(A and B) Reverse transcriptase PCR analysis: agarose gel electrophoresis of products originating from RT‐PCR reactions. In the experiments, total messenger ribonucleic acid (mRNA) isolated from human SV, penile erectile, and prostate tissue was used. The length of the fragments resulting from amplification is 656 base pairs (bp). (A) flm, fragment length (size) marker, 100 bp DNA Ladder (GeneRuler™, Fermentas GmbH); lane 1 = HCC, 1 μl MgCl_2_; lane 2 = SV, 1 μl MgCl_2_; lane 3 = SV, 2.5 μl MgCl_2_; HP = human prostate (transition zone), positive control; 0 = control. (B) RT‐PCR accuracy control, smooth muscle (sm) alpha‐actin. HCC, human corpus cavernosum penis; RT‐PCR, Reverse transcriptase polymerase chain reaction; SV, seminal vesicle.

SV tissue derived from five individuals was subjected to RT‐PCR analysis. The results seen did not provide hints to indicate that the time interval to tissue excision and freezing in liquid nitrogen might have affected in a negative manner the outcome of the amplification reactions. The accuracy of the procedures was also confirmed by the fact that the reactions were dependent on the concentration of Mg^2+^ as well as by the results of the PCR reactions on the expression of smooth muscle alpha‐actin (see Figure 1B). The experiments revealed signals related to mRNA encoding for TRPA1 in the human SV. cDNA specifically encoding for TRPA1 was present in all specimens examined. The length of the fragments resulting from the amplification was of the expected size of 656 base pairs. However, different magnitudes of mRNA expression were observed in the SV and prostate tissue, and the relative degree of expression of mRNA encoding for TRPA1, as determined by means of densitometric analysis, appeared to be much more abundant in the prostate. A typical pattern of RT‐PCR products from the experiments using human SV and prostate is depicted in Figure 1A.

### 3.2 Immunohistochemistry

**Figure 2:**
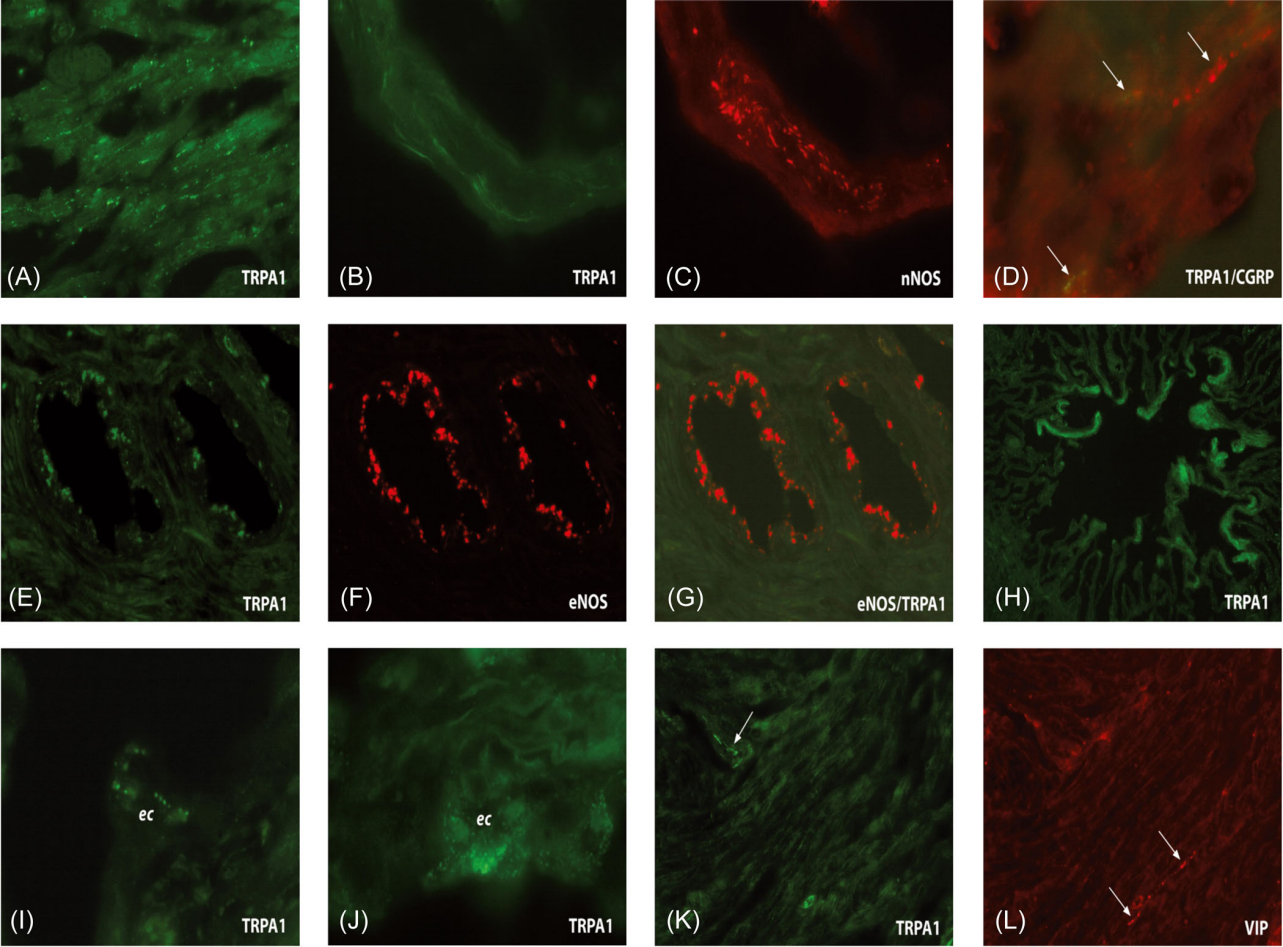
(A–L) Laser fluorescence microscopy: expression and distribution of TRPA1 in the human seminal vesicles (SVs). (A) Meshwork of slender nerve fibers expressing TRPA1 transversing the smooth muscle portion of the SV (magnification ×20). Double staining exemplifying the localization of TRPA1 (B, green) in relation to nNOS (C, red) in varicose nerve fibers captured in longitudinal orientation (magnification ×40). Localization of TRPA1 in relation to CGRP (D, marked by arrows) in single nerve fibers running through a tissue section (magnification ×40). Cross‐sectioned glandular duct presenting immunosignals for TRPA1 (E) and eNOS (F) in the secretory epithelium (magnification ×40). (G) merged image of (E) and (F). (H) Expression of TRPA1 in the epithelium of a glandular structure (magnification ×40). (I) Higher magnification of a TRPA1‐positive varicose nerve terminal close to an epithelial cell (ec) (magnification ×100, oil). (J) Higher magnification of glandular ecs exhibiting fluorescence signals specific for TRPA1 (magnification ×100, oil). (K and L) Distribution of TRPA1 (K) and the vasoactive intestinal polypeptide (VIP) (L, arrows) in varicose nerve fibers running through the smooth muscle portion of the SV (magnification ×40).

Examination of numerous sections revealed that immunofluorescence signals for TRPA1 are mainly present in slender nerve fibers transversing the smooth muscle portion of the SV as well as in the multilayer secretory epithelium of glandular ducts. Nerve fibers (some of them captured in longitudinal orientation) positive for TRPA1 were seen localized in close relation to varicose nerves containing nNOS (see Figure 2A–C). We also located TRPA1 in the glandular epithelium and in double‐stained sections, TRPA1 appeared co‐localized with the endothelial isoform of the NOS (eNOS) (see Figure 2E–H,J). The SV smooth muscle did not exhibit immunofluorescence for TRPA1. Double‐labeling with the anti‐TRPA1 and ‐CGRP or the anti‐TRPA1 and ‐VIP ABs of single slender nerve fibers running through the smooth muscle portion of the SV exhibited co‐localization of TRPA1 with CGRP, however, CGRP/TRPA1‐immunoreactive nerves were fewer than those presenting staining for nNOS and TRPA1. In contrast, no co‐localization with VIP was observed (see Figure 2D,K,L). When looking at higher magnification (×100) at the epithelial cells of cross‐sectioned glandular ducts of the SV, prominent dot‐like fluorescence signals indicated that TRPA1 is irregularly distributed throughout the cytoplasm of cell bodies of the secretory epithelium. Single varicose nerve terminals expressing TRPA1 were also noted in the epithelium (see Figure 2H,I,J). The microscopical technique used (light microscopy/laser fluorescence microscopy) did not provide hints that the immunolabeling was particularly related to subcellular structures, such as the nuclear envelope or membranes of the mitochondria, endoplasmatic reticulum or Golgi apparatus. The observations described above appeared consistent in all tissue sections examined*.*

## 4 DISCUSSION

TRPA1 holds a significant role as a cellular sensor for a large range of physical and chemical entities. In mammals, it is expressed in neurons but also other types of cells (e.g., epithelial cells, interstitial cells) and is implicated in the physiology and/or pathophysiology of the cardiovascular, respiratory and gastrointestinal systems and the urogenital tract. The channel protein is permeable to Ca^2+^, acts as a chemo‐, mechano‐ and also thermosensor, and is also involved in the local release of neuropeptides and prostanoids derived from the cyclooxygenase pathway. In addition, the role of TRPA1 in the physiological response to noxious provocation and the involvement of the ion channel in pain and inflammation emphasizes its importance in medicine. Besides being reactive to temperature, it also interacts with endogenous signaling molecules, natural compounds from plants (e.g., mustard, wasabi, garlic, horseradish, or cinnamon), and other chemicals (e.g., paraben, acrolein).^15^, ^16^, ^17^ In the human urogenital tract, where NO is an established signaling molecule, modulatory functions by NO on the activity of TRPA1 have been suggested. Furthermore, dihydrogen sulfide (H_2_S), another endogenous signaling molecule that activates TRPA1, has been reported to be involved in the regulation of functions of the lower urinary tract and cardiovascular system and the mediation of neurogenic inflammation but is also produced by invading uropathogens*.* ^18^, ^19^, ^20^ TRP channels, in particular TRPV1, TRPV2, TRPV4, TRPM8 and TRPA1, have emerged as key regulators of sensory processes in the lower urinary tract and have thus been investigated as potential targets to treat genitourinary diseases, such as the OAB, LUTS, interstitial cystitis associated with bladder disorder, chronic pelvic pain syndrome and vulvodynia.^21^, ^22^, ^23^ The increasing importance of TRP channels in research in the field of urological pharmacology has prompted us to investigate the expression and distribution of TRPA1 in human SV. Our findings demonstrate that the TRPA1 protein is mainly present in slender nerve fibers transversing the smooth muscle portion of the SV. These fibers are seen localized in close relation to varicose nerves containing nNOS and, to a lesser degree, the neuropeptide CGRP. In fact, NO has been reported to be involved in the regulation of tension in isolated SV smooth muscle.^8^ As such, it is tempting to speculate that signals mediated via TRPA1 may be linked to the control of smooth muscle tension. This has previously been described for other parts of the urogenital tract. Tissue bath studies have demonstrated that the contraction of the isolated smooth muscle of the human ureter, urethra and prostate, mediated either by alpha‐adrenergic agonists or transmural electrical stimulation, was significantly attenuated by stinging natural compounds (allyl isothiocyanate, diallyl disulfide, cinnamonaldehyde) and sodium hydrogen sulfide (NaHS), known to bind to TRPA1 and activate the channel to act as pain‐/mechanosensor at the cellular level.^10^, ^17^, ^24^, ^25^ No such functional experiments have yet been conducted using animal or human SV. Some, but not all CGRP‐positive terminals also expressed TRPA1. This may suggest that TRPA1 is expressed in subpopulations of sensory nerves. Thus, one can propose that TRPA1 may act as a detector of wall stretch and/or a sensor of the chemical composition of the SV tissue. The latter theory also relates to our finding that TRPA1 nerve varicosities extend into the epithelium. We also located TRPA1 in epithelial cells of glandular ducts that also stained for the endothelial isoform of the NOS (eNOS). NO has previously been related to the secretory functions of the SV.^26^ The location of TRPA1 in the epithelium forms a morphological basis for the involvement in secretory function. Interestingly, cinnamonaldehyde has been shown to alter both the flow rate and composition of salivary gland secretions in human volunteers.^27^ Up until today, TRPA1 has been investigated in other male and female genital tissues: In the human clitoris and vagina, TRPA1 was shown to be expressed in slender varicose nerve fibers transversing subepithelial layers. These nerves were also immunoreactive for nNOS and, in the vaginal wall, the CGRP. In clitoral epithelial cells, TRPA1 was also found co‐localized with vimentin, known as a specific feature of interstitial/neuroendocrine cells. In the human corpus cavernosum, immunoreactivity for TRPA1 was seen in nerves transversing the cavernous sinusoidal space and running alongside small penile arteries. Here, in contrast to the observations in human SV, these varicose nerves did not stain for nNOS but displayed expression of the vesicular acetylcholine transporter protein. The patterns seen in the clitoris and vagina suggest an involvement of the TRPA1 receptor in both afferent and efferent transmission, that is, in part, connected to the NO/cyclic GMP pathway.^12^, ^13^ A link between TRPA1 and (sensory) neurotransmission has also emerged from studies on tissues of the upper and lower urinary tract (ureter, urinary bladder, prostate, urethra). Tissue bath studies demonstrated that the contraction of the isolated smooth muscle of the human ureter, urethra and prostate, mediated either by alpha‐adrenergic agonists or transmural electrical stimulation, was significantly attenuated by stinging natural compounds (allyl isothiocyanate, diallyl disulfide, cinnamonaldehyde) and NaHS, known to bind to TRPA1 and activate the channel to act as pain‐/mechanosensor at the cellular level.^11^, ^18^, ^24^ In contrast, no such functional experiments have yet been conducted using animal or human SV.

In conclusion, the current study detected TRPA1 at both the transcriptional and protein level in the human SV. TRPA1 was detected at both the transcriptional and protein level in the human SV. As signals mediated by NO previously have been linked to secretory functions of the SV, one can speculate that the localization of the cationic channel in slender nerves and epithelial cells epithelium of glandular ducts form a basis for a possible role for TRPA1 in modifying secretory function. This and whether or not TRPA1 is also involved in mechano‐afferent signals and/or can modulate SV smooth muscle function by acting as an entry gate for Ca^2+^ into the cytosolic space needs to be further explored in functional settings.

## AUTHOR CONTRIBUTIONS

**Stefan Ückert**: Conceptualization; data curation; investigation; resources; supervision; writing – original draft; writing – review & editing. **Harrina E. Rahardjo**: Formal analysis; investigation; software; validation; writing – original draft; writing – review & editing. **Markus A. Kuczyk**: Project administration; resources; supervision; writing – review & editing. **Petter Hedlund**: Conceptualization; data curation; formal analysis; investigation; methodology; software; writing – original draft; writing – review & editing. All authors have read and approved the final version of the manuscript, had full access to all data from this study, and take complete responsibility for the integrity of the data and accuracy of data analysis.

## CONFLICT OF INTEREST

The authors declare no conflict of interest.

## TRANSPARENCY STATEMENT

The lead author Stefan Ückert affirms that this manuscript is an honest, accurate, and transparent account of the study being reported; that no important aspects of the study have been omitted; and that any discrepancies from the study as planned (and, if relevant, registered) have been explained.

## Data Availability

Original data from the experiments are available from the corresponding author. Previously reported bibliographic data, available at PubMed/Medline, were used to support the results of this study. These prior studies are cited within the manuscript text and listed in the references.
